# Adhesion-Induced Demolding Forces of Hard Coated Microstructures Measured with a Novel Injection Molding Tool

**DOI:** 10.3390/polym15051285

**Published:** 2023-03-03

**Authors:** Maximilian Schoenherr, Holger Ruehl, Thomas Guenther, André Zimmermann, Bernd Gundelsweiler

**Affiliations:** 1 Institute of Design and Production in Precision Engineering (IKFF), Faculty 7-Engineering Design, Production Engineering and Automotive Engineering, University of Stuttgart, Pfaffenwaldring 9, 70569 Stuttgart, Germany; 2 Institute for Micro Integration (IFM), Faculty 7-Engineering Design, Production Engineering and Automotive Engineering, University of Stuttgart, Allmandring 9b, 70569 Stuttgart, Germany; 3 Hahn-Schickard, Allmandring 9b, 70569 Stuttgart, Germany

**Keywords:** injection molding, mold tool, demolding measurement, ejection, ejection force, adhesion force, hard coating, PET

## Abstract

The demolding of plastic parts remains a challenging aspect of injection molding. Despite various experimental studies and known solutions to reduce demolding forces, there is still not a complete understanding of the effects that occur. For this reason, laboratory devices and in-process measurement injection molding tools have been developed to measure demolding forces. However, these tools are mostly used to measure either frictional forces or demolding forces for a specific part geometry. Tools that can be used to measure the adhesion components are still the exception. In this study, a novel injection molding tool based on the principle of measuring adhesion-induced tensile forces is presented. With this tool, the measurement of the demolding force is separated from the actual ejection step of the molded part. The functionality of the tool was verified by molding PET specimens at different mold temperatures, mold insert conditions and geometries. It was demonstrated that once a stable thermal state of the molding tool was achieved, the demolding force could be accurately measured with a comparatively low force variance. A built-in camera was found to be an efficient tool for monitoring the contact surface between the specimen and the mold insert. By comparing the adhesion forces of PET molded on polished uncoated, diamond-like carbon and chromium nitride (CrN) coated mold inserts, it was found that a CrN coating reduced the demolding force by 98.5% and could therefore be an efficient solution to significantly improve demolding by reducing adhesive bond strength under tensile loading.

## 1. Introduction

As the final stage of an injection molding cycle, demolding can cause damage on molded plastic parts when too-high demolding forces occur. These may result in visible ejector marks or deformed parts, which can lead to a reduced component quality as well as excessive wear on molding tools and even production downtimes due to sticking components. Demolding forces are a superposition of thermal contraction force caused by plastic shrinkage, friction force and adhesion [1]. The number of individual components varies depending on the tool design and mold part geometry. In their review, Delaney et al. [2] presented known solutions in research to reduce these individual force components.

One significant factor that influences demolding forces are the processing parameters. Shrinkage of the plastic material is particularly influential, because it determines the normal force of the plastic material acting on the mold and can therefore change the friction condition between the plastic and the cavity. Higher mold temperatures or shorter cooling times can reduce the shrinkage and therefore the necessary demolding forces, particularly in core molds [3]. Roughness also contributes to the demolding forces. In principle, higher mold roughness leads to higher demolding forces due to mechanical locking between the specimen and the mold surfaces, whereby very smooth surfaces can cause high demolding forces in certain cases as well [4]. In this case, adhesion effects become dominant as some plastics and the part begin to adhere excessively to the cavity surface. As a result, the necessary demolding forces can therefore increase for core molds with a rise in mold temperature despite lower shrinkage [5]. In addition, the roughness of machined mold components, such as core molds, has a significant impact on the force required to demold the component [6]. While draft angles or release agents may provide solutions in certain cases, they may not always provide a feasible solution due to design or application-related constraints. Hard coatings have been proposed as a means of reducing demolding forces in injection molding, since such coatings are known to reduce both frictional and adhesion forces [7,8].

Recently, studies have been conducted using molding experiments with mold building simulations for improved estimation of the demolding force [9,10]. While the results show tendencies as to how certain process parameters affect demolding, it is still not fully possible to pre-estimate demolding forces before the actual injection molding process. The relationship between adhesion effects and demolding has also not yet been adequately clarified. However, it is apparent that the choice of process parameters, as well as non-stick coatings and process parameters, has a strongly empirical character. Therefore, the demolding force for different parameters still needs to be determined via extensive testing, for which different laboratory measuring devices and in-process measurement injection molding tools have been developed.

Laboratory tools to measure static friction during demolding, which can be integrated into a tensile testing machine, were presented in [11,12].

For in-process measurement, a common approach is to use cup-, tube- or sleeve-shaped parts for specimen geometry. The plastic material is injected onto a mold core and the specimen is afterwards ejected using a stripper plate [4,13,14,15,16,17]. Due to the comparatively simple tool design, the demolding forces can be measured in a controlled and repeatable manner. The measured force is, due to the design of the mold, a superposition of frictional and adhesion-related shear strength components.

Another method of measuring demolding forces is to determine the friction between the plastic part and the tool surface by means of an opposing motion, such as sliding [7,18] or twisting [19]. To measure the pure friction between the plastic component and the tool surface, a movement of sliders in the tool is necessary before starting the actual measurement in order to decouple the plastic component from other surfaces. However, the movement of components in the mold assembly can cause vibrations, which can lead to premature detachment of adhesive bonds. This makes it difficult to consider only the adhesive force component.

A third approach is to pull a flat specimen surface vertically away from a flat mold surface [20,21]. With this method, the frictional force components can be minimized so that the measured force corresponds to the adhesive force between the plastic and the considered mold surface. These types of gauging tools also have the ability to evaluate the molding of micro-structured specimens, as they are not sheared off during demolding compared to core tools. However, flat samples suitable for producing textured surfaces are often still ejected using either one [22] or multiple ejector pins [23,24,25]. These are connected to a force sensor to measure the ejection force. In these configurations, the adhesion force between the sample surface and the mold cannot be measured directly.

In order to ensure an accurate and reliable measurement of adhesion-induced demolding forces, it is essential that the contact area between the mold and specimen does not detach before the actual measurement. Detachment can be caused by vibrations due to mechanical movements, as outlined for friction measurement tools, but also due to shrinkage of the plastic, as observed in a core tool [26]. In addition, it is desirable to monitor the contact area prior to measurement to ensure that premature detachment of the specimen does not occur. The measuring tools presented above for determining demolding forces in plastic injection molding are mainly designed to determine the frictional and shear forces that occur. Tools for determining exclusively adhesion-related demolding forces are rather rare.

In this study, a novel injection molding tool is presented that enables the measurement of adhesion-induced tensile forces during demolding, separated from a subsequent ejection of the specimen. The functionality of the injection molding tool was verified by recording the adhesive force needed to separate polyethylene terephthalate (PET) specimens from mold inserts at three mold temperatures. Two different mold geometries, either planar or structured by micro-milling, as well as uncoated and hard coated molt inserts, were tested. Since this is a completely newly developed tool, a two-factor verification was performed to ensure complete wetting of the tool surface by the plastic melt.

## 2. Materials and Methods

### 2.1. Injection Molding Tool for Adhesion-Induced Demolding Force Measurement

**Figure 1:**
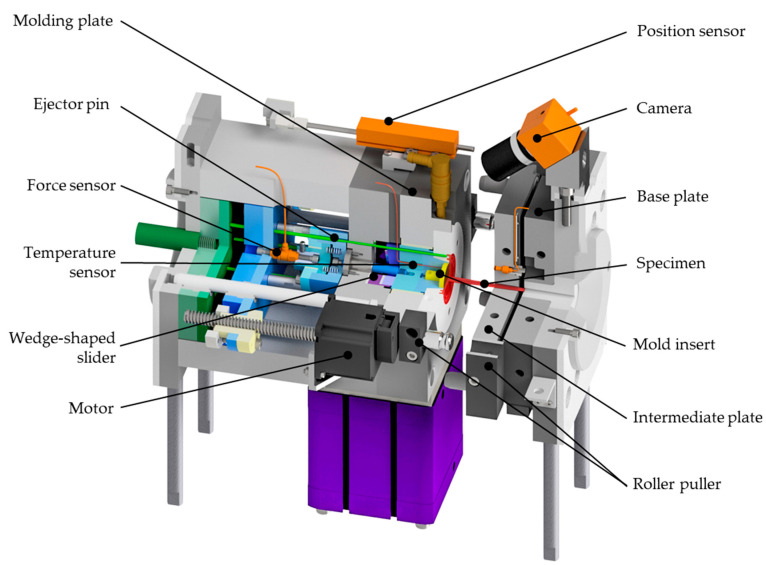
Demolding force testing tool for injection molding.

The design of the injection molding tool to measure adhesion-induced demolding forces is shown in Figure 1. The main focus during development was to ensure an intact contact surface between the specimen and a defined tool surface, which should be easily exchangeable.

**Figure 2:**
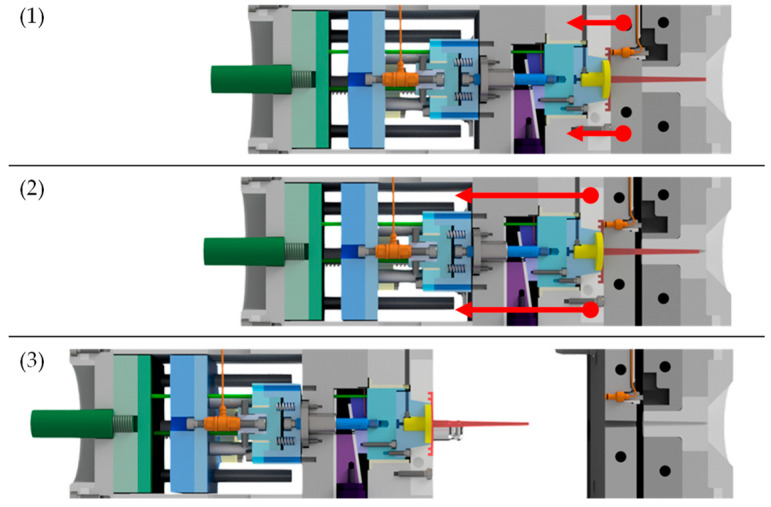
Sequence of the opening procedure: (**1**) initial position of mold plates after injection; (**2**) intermediate plate lifted from the base plate, but still in contact with the molding plate; (**3**) the tool is fully opened.

**Figure 3:**
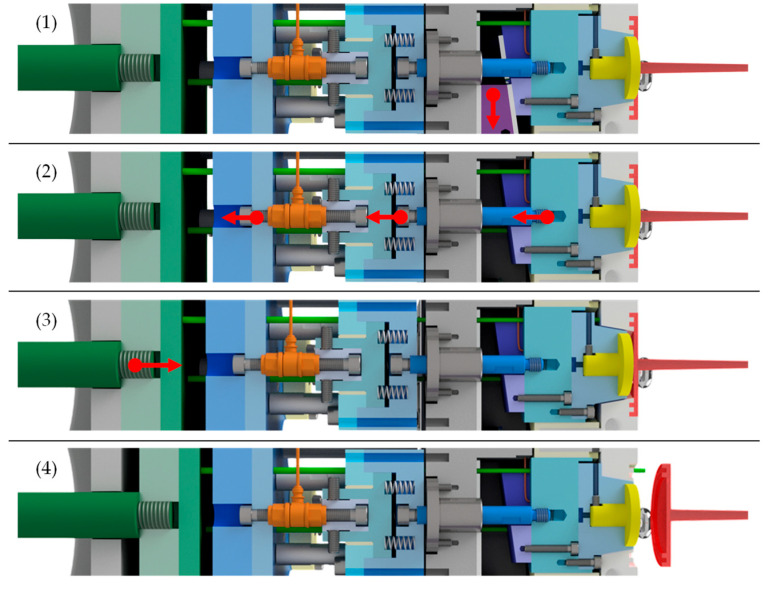
Procedure of the measuring cycle: (**1**) in the status directly after opening the mold, the wedge-shaped slider (purple) is moved downwards; (**2**) to perform the actual measurement, the measurement package (blue) is moved to the left.; (**3**,**4**) the specimen is ejected by the ejector system (green).

**Figure 4:**
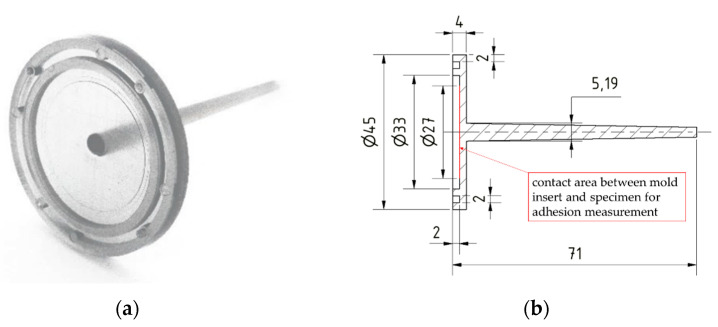
(**a**) Molded specimen and (**b**) specimen dimensions [mm].

To achieve this, the injection mold was designed as a three-plate mold with a roller puller that ensures the desired movement of the intermediate plate to prevent premature detachment of the contact surface. The specimen, red-colored in Figure 1, Figure 2 and Figure 3, was designed as a disc with double ledges on its outer circumference, as shown in Figure 4. The dimensions of the specimen were optimized by simulation to minimize warpage and thus also premature detachment [5] and are shown in Figure 4b. This ensured reliable and consistent measurements of the demolding forces. The entire thickness of the disc was 2 mm, with a total diameter of 45 mm. The melt-facing surface area of the exchangeable mold insert (MI), colored in yellow in Figure 1, Figure 2 and Figure 3, was a circular surface with a diameter of 27 mm corresponding to the contact area of the specimen. A single-axis piezoelectric force transducer 9301C (Kistler Instrumente AG, Winterthur, Switzerland), which is capable of measuringdynamic and quasi-static tensile forces up to 3 kN, was used to record the adhesion-induced demolding force. The sensor was embedded in a measuring package, blue-colored in Figure 1. Via a charge amplifier 5015A1000 (Kistler Instrumente AG, Winterthur Switzerland), the signals were fed into a measuring system. A sampling rate of 9000 Hz was used to record the demolding force. This high sampling rate ensured that the peak of the demolding force was reliably recorded. The mold cavity and measuring package were separated by a wedge-shaped slider, purple-colored in Figure 1, Figure 2 and Figure 3, to prevent the sensor cell from overload while molding. The measuring package was driven by a motor. Since mold temperature is one of the most critical process variables influencing the demolding force, a temperature sensor was used to determine the temperature directly at the mold insert before each measuring cycle. After opening the tool, a built-in camera enabled an optical detection of the contact surface between mold insert and specimen in order to verify a full wetting of the tool by the plastic melt and thus adhesion of the polymer on the mold insert. Two photos were taken per measuring cycle, one directly before and one after the measurement. The molded specimens were ejected by the use of ejector pins, green-colored in Figure 4. The individual steps per molding/measuring cycle were as follows:

(a) After closing the mold, the plasticized melt was injected into the cavity. The three plates—molding, intermediate and base plate—were initially in contact, as shown in Figure 2(1). After the mold cooled down to the desired temperature, the mold was opened. At this point, only the intermediate plate was lifted off a short distance from the base plate by the roller puller, as shown in Figure 2(2). The molding plate was still connected to the intermediate plate in this first step. This minimized mechanical stress on the contact surface of the specimen with the mold insert and prevented premature detachment.
(b) The molding plate then lifted off the intermediate plate and the tool was fully opened, as shown in Figure 2(3).
(c) The wedge-shaped slider moved downward, as shown in Figure 3(1). Via the built-in camera, the intact contact surface area between mold insert and specimen was verified.
(d) For the actual demolding force measurement, the entire measuring package was driven backwards by the motor and the mold insert detached from the specimen, as shown in Figure 3(2). The required force was recorded by the force sensor in the measuring package.
(e) The measuring package then returned to its initial position and the slider was moved up again.
(f) Finally, the specimen was ejected by the use of the ejector pins, as shown in Figure 3(3,4).

### 2.2. Mold Insert Manufacturing and Characterization

Seven mold inserts were manufactured for the injection molding experiments. All mold inserts were high-gloss polished to reduce mechanical friction to the bare minimum and thus measure solely adhesive demolding force. The melt-facing surface area of the inserts was configured to be either planar or with micro-milled channels, and either uncoated or PVD hard coated. Variation in surface conditions was investigated in order to evidence their influence on the adhesion-induced demolding force result obtained with the presented tool. The configuration of the different mold inserts is presented in Table 1.

Non-hardened hot-work tool steel 1.2343 ESR (Meusburger Georg GmbH & Co., KG, Wolfurt, Austria) was used as the raw material due to its high toughness and very good suitability to produce smooth surfaces from polishing. Using a grinder-polisher LaboPol-25 (Struers ApS, Ballerup, Denmark), the planar surfaces of the mold inserts were first plane ground and subsequently polished with a 1 µm diamond suspension in order to achieve a highly polished surface. Eighteen micro-channels were milled into the polished surfaces of MI 4, MI 5 and MI 6. The channel depth and width were set to 300 µm with a draft angle of 10°, which corresponds to the common structure dimensions of precision engineering parts, e.g., for microfluidic applications. The milling was performed using an ultra-precision milling machine Micromaster 5X-L (Kugler GmbH, Salem, Germany).

The polished and structured surfaces of MI 2 and MI 4 were then coated with a silicon modified hydrogenated amorphous (a-C:H:Si) diamond-like carbon (DLC) coating. A high-power impulse magnetron-sputtered chromium nitride (CrN) coating was deposited onto MI 3 and MI 6. Both coatings were chosen due to their high resistance to abrasive wear, the same reason these coatings are widely used in industry. The coatings were provided by industrial suppliers. MI 7 was kept in the ground surface state to compare the measured demolding force with the one measured for the CrN coated MI 3 at equivalent surface roughness values.

**Table 1:** Mold insert configuration for demolding force measurements.

Mold Insert	Pre-Condition	Structuring	PVD Hard Coating
MI 1	Polished	None	None
MI 2	Polished	None	a-C:H:Si DLC
MI 3	Polished	None	CrN
MI 4	Polished	Yes	None
MI 5	Polished	Yes	a-C:H:Si DLC
MI 6	Polished	Yes	CrN
MI 7	Ground	None	None

For a reliable comparison of the demolding forces in terms of the surface quality of the mold inserts, each mold insert was inspected prior to and after the coating deposition. The scanning electron microscope (SEM) unit of the dual beam system Helios NanoLab 600 (FEI Company, Hillsboro, OR, USA) was used to image the morphology of the mold inserts for a field of view (FOV) of 128 µm width (≙SEM magnification 1000×). Subsequently, areal surface roughness measurements were carried out with a 3D optical profiler ZYGO Nexview NX2 (Zygo Corporation, Middlefield, CT, USA). The evaluation area was set to the FOV of the SEM imaging. Further operators were set in accordance to the ISO-standard 25178-3 [27]. The arithmetic mean height (Sa), root mean square height (Sq) and maximum surface height (Sz) were determined. By this method, the topography can be directly compared with the respective morphology of the investigated mold insert. Each measurement was carried out three times on different areas on the surface and the measured roughness values were averaged. A laser probe microscope MITAKA MLP-3 (Mitaka Kohki Co., Ltd., Tokyo, Japan) was used to measure the profiles of the milled channels.

### 2.3. Injection Molding Experiments

The plastic selected for this study was PET D04 300 (DuFor Resins B.V., Zevenaar, The Netherlands). The molding temperature of PET is within the possible temperature range of the measuring mold and preliminary investigations indicated potentially difficult demolding properties for PET. In addition, PET is transparent, which enables the optical monitoring of the specimen surface adhering to the mold insert. Since these investigations also exhibited a high sensitivity of the mold temperature on the demolding force, the mold temperature was varied in the investigations. All other parameters were kept fixed. The injection molding parameters are given in Table 2.

The experiments were conducted with the injection molding machine Allrounder 270 S 250-60 (Arburg, Loßburg, Germany). Prior to the actual injection molding cycles for demolding force measurement, the molding tool was heated and then kept at the desired mold temperature for a period of 60 min. Then, 50 injection molding cycles were run, whereby only the last 15 cycles were used for the demolding force measurement.

Two-factor verification was conducted in order to confirm a complete filling of the mold cavity and thus verify complete wetting of the MI surfaces by the plasticized melt, which is required for an accurate and reliable measurement of the demolding force. After opening the mold, the built-in camera module was used to take pictures of the specimen adhering to the mold insert. Next, the areal roughness of the molded parts was measured on unstructured areas using the same measurement conditions and texture analysis operations as described for the MI in Section 2.2.

## 3. Results

### 3.1. Characterization of Mold Inserts

**Figure 5:**
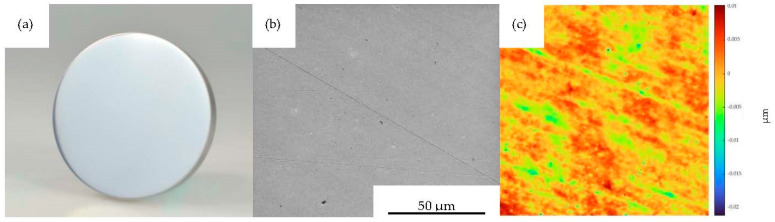
Mold insert 1; (**a**) planar uncoated mold insert, (**b**) SEM imaged surface morphology and (**c**) measured topography, both at FOV 128 µm.

For MI 1–3, mean surface roughnesses of Sa = 2.16 nm, Sq = 2.82 nm and Sz = 63.02 nm were achieved through polishing, matching optical requirements. In Figure 5a, the planar polished and uncoated MI 1 is shown. The surface is smooth, containing only a few scratches from polishing, as can be seen in Figure 5b. This is validated by the areal roughness measurement pictured in Figure 5c.

**Figure 6:**
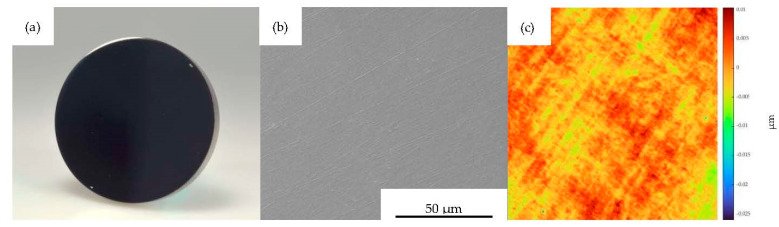
Mold insert 2; (**a**) planar DLC-coated mold insert, (**b**) SEM imaged surface morphology and (**c**) measured topography, both at FOV 128 µm.

**Figure 7:**
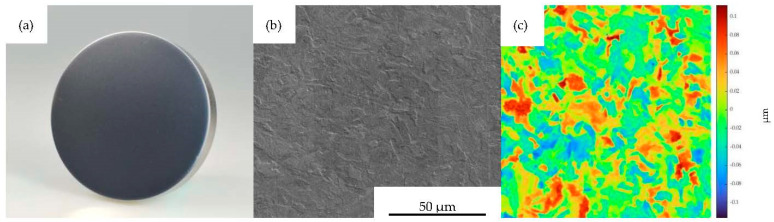
Mold insert 3; (**a**) planar CrN-coated mold insert, (**b**) SEM imaged surface morphology and (**c**) measured topography both at FOV 128 µm.

Figure 6a shows the DLC-coated planar surface of MI 2. The very low surface roughness of the polished condition was preserved. The morphology was fully reproduced by the DLC coating, as can be derived from Figure 6b,c as well as by comparing the roughness values of MI 1 and MI 2 in Table 3. In contrast, the morphology and surface roughness changed with the CrN coating. The planar surface is slightly matted (Figure 7a) and the coating morphology is of a denser, wavier texture (Figure 7b) compared to the uncoated state. Compared to the uncoated condition, for the determined surface topography in Figure 7c, the mean roughnesses were increased to Sa = 27.05 nm and Sq = 33.35 nm, as was also the case in [15]. In order to compare the subsequently conducted adhesion force measurements in terms of areal roughness, MI 7 with a mean surface roughness of Sa = 21.20 nm and Sq = 29.65 nm nearly equivalent to MI 3 was manufactured by grinding.

For MI 4–6, the surface roughnesses of unstructured areas were similar to the roughnesses of MI 1–3.

**Figure 8:**
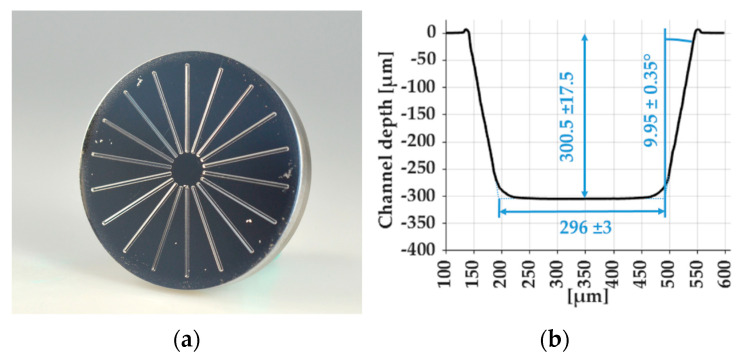
Mold insert 4: (**a**) uncoated micro-structured mold insert and (**b**) micro-channel geometry.

In Figure 8a, MI 4 is shown as an example for the micro-milled mold inserts. The profile of a micro-milled channel is shown in Figure 8b. The geometry was uniform, with a measured channel depth, width and draft angle of 300.5 ± 17.5 µm, 296 ± 3 µm and 9.95° ± 0.35.

**Figure 9:**
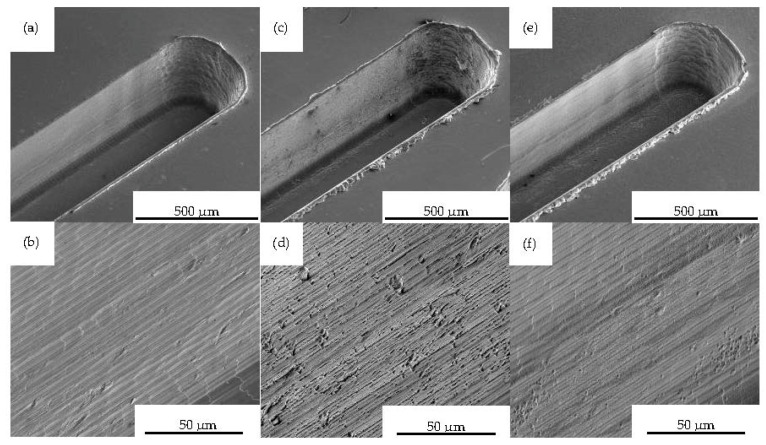
SEM imaged micro-milled channel at FOV 1020 µm and morphology of channel sidewall at FOV 128 µm of (**a**) and (**b**) MI 4, (**c**) and (**d**) MI 5, (**e**) and (**f**) MI 6.

The draft angle of MI 4 was steep-stepped, as can be derived from Figure 9a. However, tool marks on the draft angle and some smaller material breakouts that occurred during machining were found, as can be seen in Figure 9b.

The SEM-imaged coated micro-channels, as well as their morphology, are shown in Figure 9c–f. Over the entire mold insert surface, the deposited coatings were uniform. The micro-channel sidewalls were also coated. However, the coating contained micro-droplets, resulting in a craggy morphology. Due to the steepness of the sidewalls, the areal roughness could not be determined by using optical measurement devices.

### 3.2. Injection Molding Tool Functionality

#### 3.2.1. Typical Force Curve for a Demolding Force Measurement

**Figure 10:**
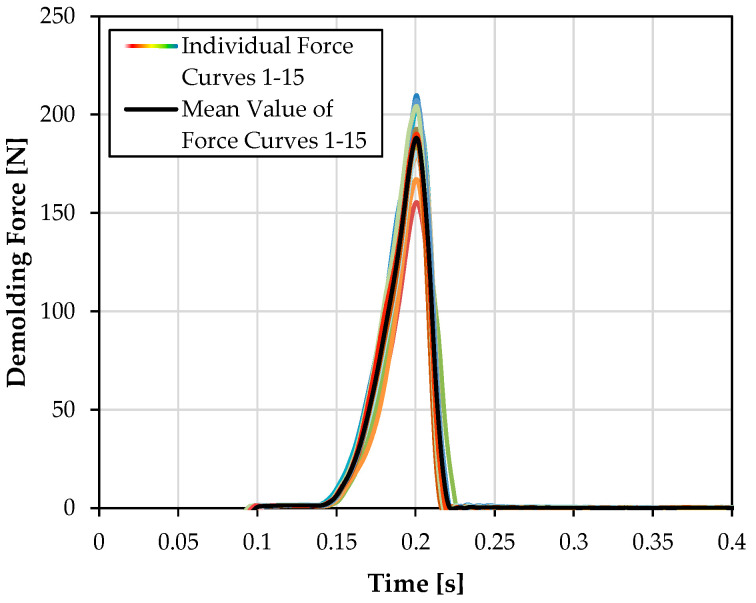
Typical force curve of the measuring tool. Fifteen consecutive measurements (color) and their mean value (black) using an uncoated mold insert at a mold temperature of 40 °C.

A typical force curve, which was recorded with the presented injection molding tool, is shown in Figure 10. The last 15 measurements of the 50-cycle test are printed in different colors on top of each other. The mean value of the measured curves is presented in black color. All force curves show an equal curve progression. The variance of the force peak in the measurements is in the range from 10 to 20%. Blank measurements as well as the force profile show force values of below 1 N immediately after the detachment process. Parasitic force values, such as those caused by mechanical movement of the measuring package, are thus small compared to the demolding force values and were neglected for further measurements.

#### 3.2.2. Stability of Measurements

**Figure 11:**
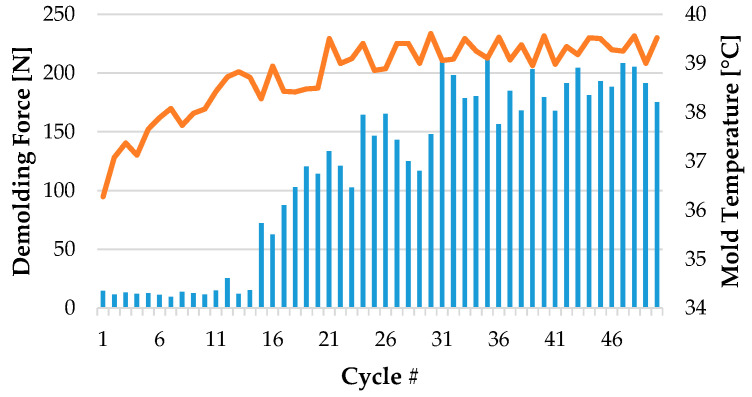
Typical progression of temperature (orange) and measured forces (blue) over the injection molding cycles.

**Figure 12:**
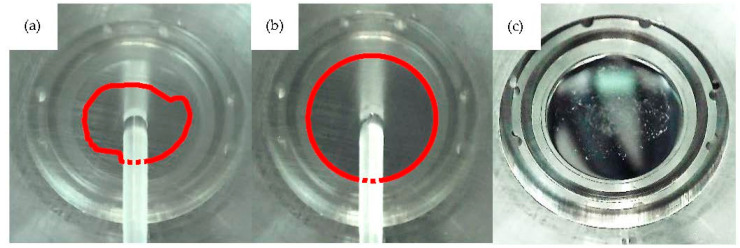
Adhesive sticking of the mold surface (**a**) after the 3rd cycle and (**b**) after the 30th cycle. Deposits on the mold insert after ejection of the specimen are shown in (**c**).

The evaluation of 50 consecutive measurement cycles is shown in Figure 11. The demolding force corresponded in each case to the force peaks from the measurement. In principle, the course can be divided into three phases:

1. Within the first 15 cycles, contaminants such as oil and grease were still present on the surface. A low level of wetting was also observed at the beginning of the measurements (Figure 12). The determined demolding force was therefore very low.
2. In the next 15 measuring cycles, the demolding force increased steadily due to an established thermal equilibrium caused by the heat supply through the melt. Any contaminants that may be present were usually removed by the 30th cycle. In certain measurements, increasing amounts of polymer residues on the mold insert were also observed.
3. After approximately 30 cycles, the system was in a steady state. The measured demolding forces did not increase any longer.

This procedure was performed before each measurement to achieve a steady state for comparable results.

#### 3.2.3. Optical Monitoring of the Contact Surface

A full wetting of the mold insert by the plasticized melt, resulting in a full adherence of the specimen surface to the mold insert, is required to measure the adhesion-induced demolding force. For transparent plastics, the surface area of the specimen adhering to the MI surface can be monitored with the built-in camera tool, which was used as an initial verification step.

Figure 12a shows the contact area between a specimen and a mold insert at the beginning of the molding cycles. Compared to Figure 12b, which shows the state after the injection molding tool has run into a steady state, it can be derived that initially, only partial wetting of the MI surface by the polymer melt occurs. As soon as the process was in a thermal steady state at the 30th molding/measurement cycle, a full wetting of the mold insert surface was achieved. As shown in Figure 12c, polymer residuals were observed for both the uncoated MI 1 and the DLC coated MI 2 after a few cycles. The amount of residuals, however, increased with each cycle until a stable condition was finally achieved after approximately 10–20 cycles.

### 3.3. Measurement of Demolding Forces for Mold Inserts

#### 3.3.1. Planar Mold Inserts MI 1–MI 3

**Figure A1:**
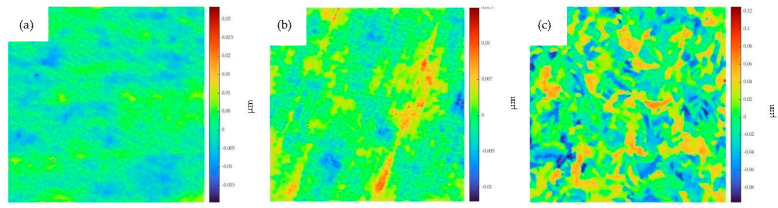
Surface topographies of injection-molded specimens at demolding temperature 60 °C for (**a**) MI 1, (**b**) MI 2 and (**c**) MI, all measured for FOV 128 µm.

For the mold inserts MI 1–MI 3 and each mold temperature, the surface roughness was measured as a second verification step to confirm replication fidelity. Both the surface roughness values of the replicated parts and the surface morphologies exemplarily evaluated for a mold temperature of 60 °C are shown in Figure A1 and Table A1. The well-replicated mold insert morphologies on the plastic parts, in combination with the well-matching roughness values of the specimens with the roughnesses given in Table 3, verify a high replication fidelity for the investigated injection molding parameters with this tool.

**Figure 13:**
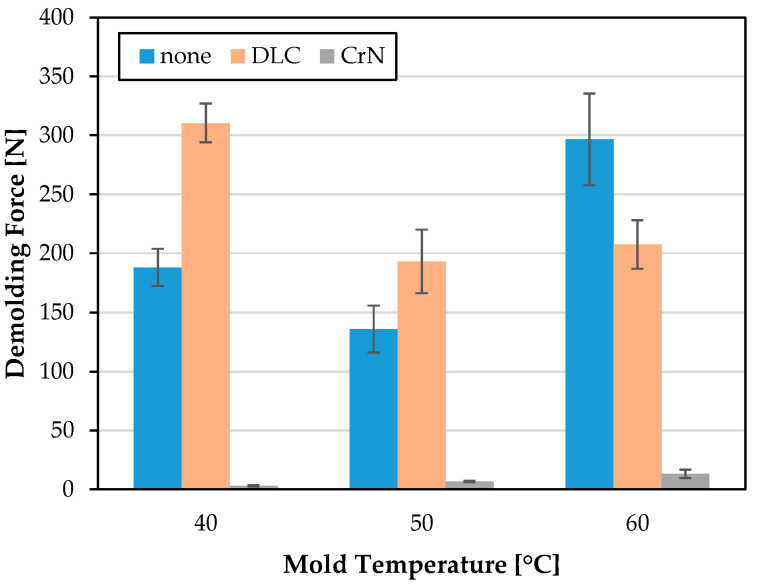
Demolding force [N] of PET for planar, uncoated, DLC coated and CrN coated MI 1, MI 2 and MI 3 at mold temperatures 40 °C, 50 °C and 60 °C.

The results of the demolding force measurements of the PET for the planar mold inserts MI 1–MI 3 are shown in Figure 13. While the demolding forces for MI 1 and MI 2 show average maximum forces in the range of 135 to 310 N, the CrN-coated mold insert shows consistently significantly lower forces up to an average maximum of merely 13 N. The required demolding force increases with rising mold temperature. In contrast, both the uncoated and the DLC-coated mold insert show the lowest demolding force at 50 °C. At a mold temperature of 60 °C, a DLC coating is advantageous, whereas at lower temperatures, an uncoated mold temperature offers better demolding force behavior compared with a DLC coating.

**Figure 14:**
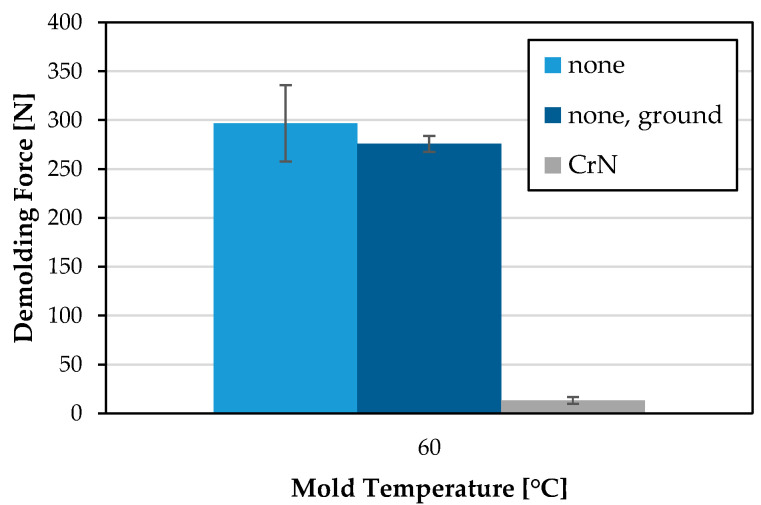
Demolding force [N] of PET for planar polished, planar ground and planar CrN coated MI 1, MI 3 and MI 7 at mold temperature of 60 °C.

Since the CrN coated MI 3 shows significantly lower demolding forces (−98.5% at 40 °C and −95.6% at 60 °C), but also a higher roughness, which, in principle, has an influence on the demolding result with other measuring tools [4], the uncoated mold insert MI 7 was ground to an equivalent roughness. A comparative injection molding was carried out at a mold temperature of 60 °C, for which the highest difference in the demolding force was found between MI 1 and M3. The results are presented in Figure 14. The slight increase in surface roughness leads to a slightly lower demolding force. However, the demolding force of the uncoated MI 7 is still significantly higher compared to MI 3 with equivalent roughness.

#### 3.3.2. Micro-Milled Mold Inserts MI 4–MI 6

**Figure 15:**
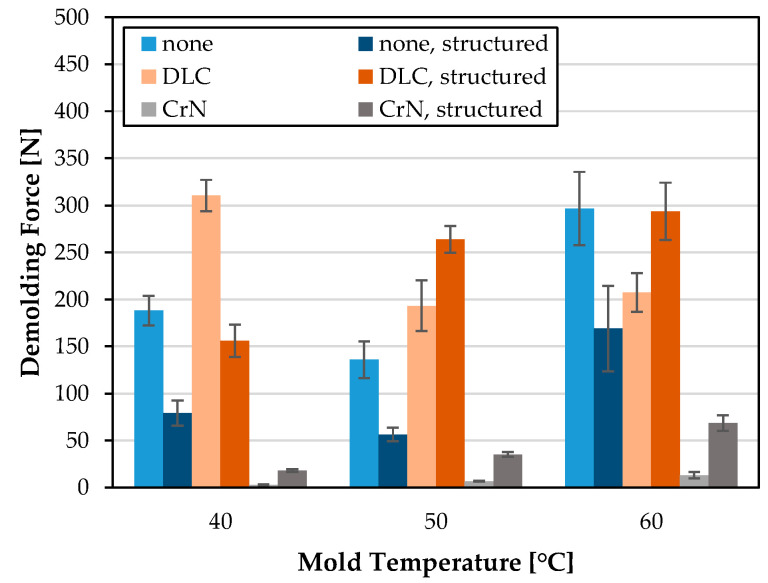
Demolding force [N] of PET for planar, uncoated, DLC coated and CrN coated MI 1, MI 2 and MI 3 (bright colored) compared to structured, uncoated, DLC coated and CrN coated MI 4, MI 5 and MI 6 (dark colored) at mold temperatures 40 °C, 50 °C and 60 °C.

The influence of the channel structures, micro-milled into the mold inserts MI 4–MI 6, on the measured demolding force seems to be very inconsistent, as shown in Figure 15. For MI 4, the structuring led to significantly lower demolding forces, irrespective of the mold temperature. In contrast, the CrN coated MI 3 and MI 6 exhibited the opposite behavior. The microstructures led to an increased demolding force for all investigated mold temperatures. With the DLC-coated mold inserts, the microstructures led, in some cases, to a drastic reduction in the demolding force (40 °C) or increased the demolding force (50/60 °C).

When comparing the structured mold inserts, a similar result was obtained to the comparison of the unstructured mold inserts. The highest demolding forces of the structured mold inserts were measured for MI 5, which are higher than the uncoated MI 4 over the entire temperature range. For MI 6, a significantly lower demolding force compared to MI 4 or DLC coated MI5 was found for all investigated temperatures. Especially at a mold temperature of 60 °C, a high absolute demolding force reduction can be observed. In comparison to the uncoated MI 4, the demolding force of MI 6 decreased by an average of 92.7 N (−59.6%). In comparison to MI 5, the demolding force decreased by an average of 225.2 N (−76.7%). At the low temperatures investigated, a very high relative decrease in the demolding force was observed. In this case, the decrease was −77.1% compared to MI4 and −88.4% compared to DLC coated MI 5.

The results were gained on the premise that a full wetting of the MI 4–MI 6 and thus a high replication fidelity could be determined for the investigated molding parameters.

## 4. Discussion

In this work, a novel type of injection mold was presented that was designed to investigate purely adhesion-related demolding forces between plastics and the mold surface. After running in the injection molding tool, a steady state of the demolding force peak with a variance within a range of 20% was achieved. Since comparable studies largely do not provide information on the measurement variance, a comparison among different measurement concepts is difficult. Core tools that do not solely measure adhesion-induced demolding forces exhibited a high variance of up to >40% [15,26]. In this context, a sufficient reliability of the measurement results can be attested.

The two-step verification conducted shows that the required wetting of the mold insert surface by the plasticized melt and thus a full adherence of the specimens to the inserts can be achieved with the presented molding tool. For future measurements, the usage of the built-in camera should be sufficient without the need for additional roughness measurements.

In accordance with previous investigations, the results for the adhesion-induced demolding forces show a strong dependence of the demolding force on the coatings [14,28], the mold insert design [15] and surface roughness [29]. For PET, the CrN coating has a positive influence on the demolding behavior compared to an untreated surface or a DLC coated surface, regardless of incorporated structures or the surface roughness. The high demolding forces occurring with planar, uncoated mold inserts cannot be explained solely by the slightly lower surface roughness compared to CrN, as the investigations with ground mold inserts show. According to [30], there is a region with minimal deformation force. As also found by Tillmann et al. in [15], coating with CrN increases surface roughness, which resulted in an increase in deformation force. Yet, a core tool was used for measurement in this study. The significant reduction in measured forces with CrN observed in this study suggests that the direction of deformation has a significant impact on the performance of coatings and, therefore, further research is necessary.

However, in the case of unstructured mold inserts MI1 and MI 2, some PET residues were found on the mold inserts after a few injection molding cycles, which was not the case for the CrN-coated MI 3 and MI 7. Such deposits indicate a strong adhesion of plastic material to the mold surface and a cohesive failure in the specimen, which results in high forces required to separate the part. These effects cannot be solely used to fully explain the results for MI 1 and MI 2 as well as MI 4 and MI 6, showing a minimum demolding force at 50 °C. In contrast, a slight and steady increase in the demolding force was observed for CrN with increasing mold temperature. The presented results are in accordance with previous studies, which also present a volatile influence of the mold temperature on the demolding for different plastic materials [22,23]. However, adhesion is not only related to the temperature itself but also to the chemical affinity of two adjacent materials [2] as well as their individual thermal properties [8].

A positive influence on the demolding force could be the roughening of the mold insert surfaces [4]. This means that milled microstructures, which are of higher roughness than polished surfaces, can have a positive influence on demolding behavior under certain conditions. However, under very low demolding forces, as occurred with the CrN coated mold inserts, the microstructures had a negative effect on the demolding behavior. In this case, other effects, such as mechanical anchoring of the plastic to the fine ridge of the microstructures, might dominate.

## 5. Conclusions

In this study, a novel injection molding tool with an integrated demolding force measurement of adhesion-induced tensile forces, separated from the ejection of the specimen, was presented. The study can be summarized as follows:

- The presented tool is shown to be effective in measuring adhesion-induced demolding forces with a comparatively good variance.
- A two-factor verification was helpful to ensure complete wetting of the tool surface by the plastic melt. For further investigations, the use of the built-in camera should be sufficient to monitor the wetting, at least of amorphous plastic materials.
- To verify the function of the tool, PET specimens were molded on both uncoated and hard coated mold inserts and then demolded at mold temperatures of 40 °C, 50 °C and 60 °C. CrN provided the lowest demolding force for the experimental setup, regardless of whether the mold insert surface was planar or structured. A complete explanation of how molding parameters and plastic and mold material properties affect the adhesive demolding force in detail cannot yet be given.

For future research, we plan to use the presented tool to investigate plastics and coating materials for varying molding parameters further to gain a deeper understanding on demolding behavior, including related effects.

## Figures and Tables

**Table 2:** Molding parameters used in this study.

Parameter	
Mold temperature [°C]	40, 50, 60
Injection speed [cm^3^/s]	15
Injection pressure [bar]	500
Measuring package speed [mm/s]	5
Molding temperature [°C]	280

**Table 3:** Surface roughness of mold inserts no. 1, 2, 3 and 7.

Mold Insert	Sa [nm]	Sq [nm]
1	2.15	2.70
2	2.00	2.50
3	27.05	33.35
7	21.20	29.65

## Data Availability

Data presented in this study are available on request from the corresponding author.

## Appendix A

**Table A1:** Surface roughness of injection-molded parts from mold inserts MI 1–3.

Mold Insert	Mold Temperature [°C]	Sa [nm]	Sq [nm]
	40	2.30	2.89
MI 1	50	2.25	2.87
	60	2.37	2.98
	40	2.05	2.64
MI 2	50	2.24	2.85
	60	2.12	2.71
	40	25.09	30.59
MI 3	50	25.90	32.09
	60	26.20	32.30
